# Genome-wide association with diabetes-related traits in the Framingham Heart Study

**DOI:** 10.1186/1471-2350-8-S1-S16

**Published:** 2007-09-19

**Authors:** James B Meigs, Alisa K Manning, Caroline S Fox, Jose C Florez, Chunyu Liu, L Adrienne Cupples, Josée Dupuis

**Affiliations:** 1 General Medicine Division, Department of Medicine, Massachusetts General Hospital and Harvard Medical School, Boston, MA, USA; 2 Department of Biostatistics, Boston University School of Public Health, Boston, MA, USA; 3 The Division of Endocrinology, Diabetes, and Hypertension, Brigham and Women's Hospital, Harvard Medical School, Boston, MA, and the National Heart, Lung, and Blood Institute's Framingham Heart Study, Framingham, MA, USA; 4 Diabetes Unit, Department of Medicine and Center for Human Genetic Research, Massachusetts General Hospital and Harvard Medical School, Boston, MA, and Program in Medical and Population Genetics, Broad Institute of Harvard and MIT, Cambridge, MA, USA

## Abstract

**Background:**

Susceptibility to type 2 diabetes may be conferred by genetic variants having modest effects on risk. Genome-wide fixed marker arrays offer a novel approach to detect these variants.

**Methods:**

We used the Affymetrix 100K SNP array in 1,087 Framingham Offspring Study family members to examine genetic associations with three diabetes-related quantitative glucose traits (fasting plasma glucose (FPG), hemoglobin A1c, 28-yr time-averaged FPG (tFPG)), three insulin traits (fasting insulin, HOMA-insulin resistance, and 0–120 min insulin sensitivity index); and with risk for diabetes. We used additive generalized estimating equations (GEE) and family-based association test (FBAT) models to test associations of SNP genotypes with sex-age-age^2^-adjusted residual trait values, and Cox survival models to test incident diabetes.

**Results:**

We found 415 SNPs associated (at p < 0.001) with at least one of the six quantitative traits in GEE, 242 in FBAT (18 overlapped with GEE for 639 non-overlapping SNPs), and 128 associated with incident diabetes (31 overlapped with the 639) giving 736 non-overlapping SNPs. Of these 736 SNPs, 439 were within 60 kb of a known gene. Additionally, 53 SNPs (of which 42 had r^2 ^< 0.80 with each other) had p < 0.01 for incident diabetes AND (all 3 glucose traits OR all 3 insulin traits, OR 2 glucose traits and 2 insulin traits); of these, 36 overlapped with the 736 other SNPs. Of 100K SNPs, one (rs7100927) was in moderate LD (r^2 ^= 0.50) with *TCF7L2 *(rs7903146), and was associated with risk of diabetes (Cox p-value 0.007, additive hazard ratio for diabetes = 1.56) and with tFPG (GEE p-value 0.03). There were no common (MAF > 1%) 100K SNPs in LD (r^2 ^> 0.05) with *ABCC8 *A1369S (rs757110), *KCNJ11 *E23K (rs5219), or SNPs in *CAPN10 *or *HNFa*. *PPARG *P12A (rs1801282) was not significantly associated with diabetes or related traits.

**Conclusion:**

Framingham 100K SNP data is a resource for association tests of known and novel genes with diabetes and related traits posted at http://www.ncbi.nlm.nih.gov/projects/gap/cgi-bin/study.cgi?id=phs000007. Framingham 100K data replicate the *TCF7L2 *association with diabetes.

## Background

Type 2 diabetes is a cause of poor health and early death that is spreading worldwide and exerting a fearsome human and economic toll [1,2]. Prevention and control of diabetes requires a better understanding of its basic molecular causes. Type 2 diabetes is a heterogeneous disease arising from physiological dysfunction in the pancreas, skeletal muscle, liver, adipose and vascular tissue. Much of the heterogeneity of type 2 diabetes has a genetic basis. A full picture of the complex genetic architecture of diabetes has been elusive [3-7].

Among type 2 diabetes susceptibility genes few, if any, individual loci are expected to carry alleles of major effect explaining a substantial proportion of cases, although a few genes could have a substantial population effect but not give a strong genetic signal if the causal alleles were common and the increase in risk were modest [6,7]. Such genes have proven hard to detect using linkage-based approaches, although recent rapid advances in genetic association methodologies have led to some successes. The P12A polymorphism in the gene encoding the peroxisome proliferator-activated receptor-g (*PPARG*) [7], the E23K polymorphism in the gene encoding the islet ATP-dependent potassium channel Kir6.2 (*ABCC8*-*KCNJ11*) [8-10] and common variants in the gene encoding the transcription factor 7-like 2 gene (*TCF7L2*) [11,12] were all found using well-powered association mapping, and all have been reproducibly associated with diabetes in diverse samples at highly significant p-values.

Current gene discovery strategies have focused on coding regions, but regulatory variants also influence disease [11,13,14]. A comprehensive picture of diabetes genetics will require a wide and adequately dense search across coding and conserved non-coding genomic regions using an association analysis approach, where power is superior to linkage analysis when seeking common variants of modest effect [6]. Resources are now becoming available to perform such genome-wide association (GWA) studies of type 2 diabetes [15-18].

In this report we describe the Framingham Heart Study (FHS) Affymetrix 100K SNP genome-wide association (GWA) study resource for type 2 diabetes. This resource complements the several other large extant type 2 diabetes GWA studies in three major respects: it is population-based (not diabetes proband-based), studies two generations, and has decades of longitudinal, standardized, detailed follow-up. We describe results of a simple low p-value-based SNP selection strategy and an alternate novel SNP selection strategy that takes advantage of the unique FHS diabetes-related quantitative traits data. We use FHS 100K SNPs in an *in silico *replication analysis that tests the hypothesis that SNPs in LD with published causal variants in *PPARG*, *ABCC8*, *TCF7L2*, *CAPN10*, and *HNFa *are associated with diabetes and related quantitative traits.

## Methods

### Study subjects

The study sample is described in the Overview *Methods *section [19]. With respect to diabetes-related traits, Offspring subjects provided genotypes and diabetes-related traits to the analyses, and Offspring parents from the Original FHS Cohort contributed genotypes for linkage analysis and FBAT statistics. Of 1,345 FHS subjects with 100K SNP data, 1,087 were Offspring and of these 560 were women, the mean age at exam 5 was 52 years, and the mean age at last follow-up was 59 years. Every study subject provided written informed consent at every examination, including consent for genetic analyses, and the study was approved by Boston University's Institutional Review Board.

### Genotyping and annotation

Affymetrix 100K SNP and Marshfield STR genotyping are described in the Overview *Methods *section [19]. Genotype annotation sources are described in the Overview *Methods *section [19].

### Diabetes phenotyping

Diabetes and related quantitative traits have been ascertained at every FHS exam for every generation. Diabetes-related quantitative traits available in the FHS 100K resource are displayed in Table 1. FPG data for the analyses came from all 7 Offspring exams, but the remainder of the data came from exam 5 (1991–94), when subjects without diagnosed diabetes underwent a 75 gram oral glucose tolerance test, or exam 7 (1998–2001), the most recent exam. We defined diabetes as chart-review-confirmed diabetes, new or ongoing hypoglycemic treatment for diabetes at any exam, or a FPG > 125 mg/dl at two or more of the seven exams. Diabetes age-of-onset was defined as the subject's age at the exam at which diabetes was first identified. Among Offspring with diabetes, >99% have type 2 diabetes [4]. Of the 1,083 Offspring with 100K genotypes and known diabetes status, 91 had diabetes. The mean age of onset of was 58 yr; through exam 7, 9.3% of diabetic subjects had developed diabetes by age 40 yr, 33.0% by age 50, 68.1% by age 60, and 99.7% by age 80.

**Table 1:** Type 2 diabetes-related quantitative traits in 1087 Framingham Offspring Study subjects with 100K genotype data

Trait	Number of traits	Offspring Exam Cycle	Cohort Exam Cycle	Adjustment *	Number with Genotype and Trait Levels †
Fasting plasma glucose (FPG)	1	5, 7	-	age, age^2 ^age, age^2^, BMI	1,027
Hemoglobin A1c (HbA1c)	1	5, 7	-	age, age^2 ^age, age^2^, BMI	623
28 yr time averaged FPG (tFPG)	1	1–7	-	age, age^2 ^age, age^2^, BMI	1,087
Fasting insulin	1	5, 7	-	age, age^2 ^age, age^2^, BMI	982
Homeostasis model insulin resistance (HOMA-IR)	1	5	-	age, age^2 ^age, age^2^, BMI	980
0–120 min insulin sensitivity (ISI_0-120)	1	5	-	age, age^2 ^age, age^2^, BMI	935
Incident type 2 diabetes	1	1–7	-	age, age^2 ^age, age^2^, BMI	91 with diabetes 1,083 without diabetes
Adiponectin	1	7	-	age, age^2 ^age, age^2^, BMI	828
Resistin	1	7	-	age, age^2 ^age, age^2^, BMI	831

* Traits were modeled as log(trait value) in sex-specific models. Residuals from these models were tested as quantitative traits associated with SNP genotype, and ranked residuals were used in linkage analyses.

† For traits with data at both exams 5 and 7, numbers are given for subjects with data at exam 5

In this presentation we focus on six (three glucose and three insulin) primary Offspring diabetes-related quantitative traits. Glucose traits are fasting plasma glucose (FPG) and hemoglobin A1c (HbA1c) measured at exam 5, and up to 28 yr time-averaged FPG (tFPG) level obtained from the mean of up to seven serial exams. Glucose traits included all subjects, including those with diabetes regardless of treatment, as these were the most informative subjects with respect to hyperglycemia. Subjects with diabetes had the highest glucose values when subjects were ranked with respect to any glucose trait; those on treatment had the highest values. The three insulin traits are fasting insulin, homeostasis model-assessed insulin resistance (HOMA-IR), and Gutt's 0–120 min insulin sensitivity index (ISI_0-120) measured at exam 5. Subjects with insulin-treated diabetes were removed from all insulin trait analyses, as we had no information on insulin dose and so measured insulin values were confounded by insulin treatment [20-22]. We also analyzed incident diabetes from first exam through last follow-up. We previously have described FHS laboratory methods for these diabetes-related quantitative traits [4,23-25]. In addition to glucose and insulin traits, levels of adiponectin and resistin are available in the FHS dbGaP resource. Plasma adiponectin and resistin concentrations were measured using a commercial ELISA (R&D Systems, Minneapolis, MN); inter- and intra-assays CVs were 5.3%–9.6% for adiponectin and 7.6%–10.5% for resistin.

### SNP prioritization

We used two approaches to prioritize SNPs potentially associated with diabetes or diabetes related traits. In the first, we simply ordered SNPs from lowest to highest p-value for association with one or more of the six primary glucose and insulin traits. We also ordered SNPs or Marshfield STRS by highest to lowest LOD score for linkage to one or more of the six primary traits, and present LOD scores > 2.0. In an alternative SNP prioritization strategy, we selected SNPs associated with multiple-related traits. In this approach, we selected SNPs with consistent nominal associations (p < 0.01 in GEE or FBAT) with all three glucose traits OR all three insulin-related traits OR (two glucose and two insulin traits). Among these we used extent of LD to select a non-redundant set of SNPs; when several were perfect proxies for each other (r^2 ^≥ 0.8) only one SNP was selected, based on the highest genotyping call rate.

### Statistical analysis

The general statistical methods for linkage and GWA analyses are described in the Overview *Methods *[19]. For diabetes-related quantitative traits we used additive GEE and FBAT models, testing associations between SNP genotypes and age-age^2^-sex-adjusted residual trait values. We kept 70,987 SNPs in the analyses that were on autosomes, had genotypic call rates ≥ 80%, HWE p ≥ 0.001 and MAF ≥ 10%.

We tested association of 100K SNPs with incident type 2 diabetes in two additional models using the same adjustment strategy. First, Martingale residuals were created to measure the age-of-onset of type 2 diabetes; residuals were analyzed with FBAT [26]. Individuals with lower values of this 'martingale residual' trait developed diabetes at younger ages, and those with the highest values had been observed for the longest time without development of diabetes [27]. Second, we used a Cox proportional hazard survival analysis with robust covariance estimates in order to find SNPs associated with development of diabetes over all seven exams [28].

## Results

Diabetes-related quantitative traits available in the FHS 100K SNP resource are listed in Table 1 and posted on the NCBI web site [29]. Each trait is available as an age-age^2^-adjusted or age-age^2^-BMI-adjusted residuals from sex-specific models. In this analysis we only consider the age-age^2^-adjusted traits. Among these, the following were the primary traits used in this analysis: exam 5 fasting plasma glucose (FPG; n with data = 1,027; mean, SD 99, 24.7 mg/dl); exam 5 HbA1c (n = 623; 5.28, 0.9%); 28-year time averaged FPG (tFPG; n = 1,087; 98, 16.2 mg/dl); exam 5 fasting insulin (n = 982; 30.1, 16.4 uU/ml); exam 5 HOMA-IR (n = 980; 7.8, 7.3 units); and the 0–120 min insulin sensitivity index (ISI_0-120; n = 935; 26.1, 7.6 mg·l^2^/mmol·mU·min). Among 1,087 Offspring with 100K SNP data there were 91 cases of type 2 diabetes. Additional diabetes-related quantitative traits not used in this analysis but that are available in the FHS 100K SNP dbGaP resource include, at exam 7: FPG (n = 987; 103, 26 mg/dl); fasting insulin (n = 999; 15.8, 12.8 uU/ml); HOMA-IR (n = 969; 4.2, 4.1 units); HbA1c (n = 893; 5.59, 0.97%); resistin (n = 831; 14.5, 7.4 ng/dl); adiponectin (n = 828; 9.9, 6.2 ng/dl).

The six primary quantitative traits had significant associations with 415 SNPs in GEE models and 242 SNPs in FBAT models, using p-value < 0.001, and only considering SNPs with call rate ≥ 0.80, HWE p-value ≥ 0.001, and MAF ≥ 10%. Additionally, there were 91 significant associations with incident diabetes in the survival analyses and 42 significant associations with age-of-onset in FBAT, representing 128 non-overlapping SNPs. The 25 SNPs with lowest p-values in GEE or FBAT models, and LOD scores > 2.0 in linkage analyses, are displayed in Table 2. After accounting for the overlap between sets of significant associations, 736 non-overlapping SNPs were identified by the p-value approach for SNP prioritization.

**Table 2:** Twenty five lowest p-values from GEE and FBAT models and LOD scores > 2 for 100K SNPs and FHS diabetes-related quantitative traits

No.	Trait	SNP	Chr	Physical position	GEE or Cox p-value	FBAT p-value	Known Genes	
**2a. Ordered by GEE p-value**								

1	tFPG	rs2722425	8	40603396	0.00000002	0.0047	ZMAT4	
2	Incident DM	rs10497721	2	192739868	0.0000007	0.0346	TMEFF2	
3	Fasting Insulin	rs2877832	14	26870017	0.000002	0.0770		
4	HOMA-IR	rs2877832	14	26870017	0.000003	0.0918		
5	tFPG	rs10510634	3	30321972	0.000005	0.0516		
6	FPG	rs180730	4	122159395	0.000005	0.0374	PRDM5	
7	tFPG	rs180730	4	122159395	0.000006	0.0252	PRDM5	
8	tFPG	rs7731657	5	129971218	0.000007	0.0015		
9	HbA1c	rs10486607	7	28957729	0.000008	0.0440	CPVL	
10	ISI_0-120	rs2066219	13	68428665	0.000009	0.0245		
11	FPG	rs2722425	8	40603396	0.000009	0.0998	ZMAT4	
12	Incident DM	rs2195499	2	41778050	0.000011	0.3860		
13	Incident DM	rs830604	3	71673037	0.000014	0.5914	FOXP1	
14	FPG	rs2377689	2	106924358	0.000017	0.0015	ST6GAL2	
15	Incident DM	rs931567	3	31410581	0.000018	0.0095		
16	FPG	rs10494331	1	156395176	0.000019	0.0369	APCS	
17	tFPG	rs7147624	14	64935378	0.000019	0.0201	FUT8	
18	tFPG	rs931567	3	31410581	0.000019	0.0189		
19	Incident DM	rs10511182	3	102255525	0.000020	0.0510		
20	FPG	rs337112	5	122556671	0.000022	0.0148		
21	ISI_0-120	rs9319109	13	84510270	0.000025	0.0048		
22	HOMA-IR	rs1927384	13	101943751	0.000026	0.0059		
23	ISI_0-120	rs7139897	13	107879625	0.000026	0.0033		
24	HbA1c	rs721346	11	103242667	0.000027	0.5054	PDGFD	
25	HOMA-IR	rs300703	2	229416	0.000027	0.1086	SH3YL1	

**2b. Ordered by FBAT p-value**								

1	HbA1c	rs7719971	5	119990475	0.0324	0.00002		
2	HOMA-IR	rs10425253	19	36038375	0.0005	0.00002		
3	HOMA-IR	rs10511886	9	31826555	0.0933	0.00002		
4	Incident DM	rs256962	5	114970610	0.0030	0.00002	TICAM2	
5	Fasting Insulin	rs10494321	1	154721517	0.0029	0.00002	KIRREL	
6	Incident DM	rs1549415	8	120252290	0.0332	0.00002		
7	FPG	rs6910169	6	112990680	0.0062	0.00003		
8	HbA1c	rs2400207	5	145360290	0.0305	0.00003	SH3RF2	
9	HbA1c	rs991672	5	120002649	0.0272	0.00003		
10	ISI_0-120	rs633082	9	107992360	0.0068	0.00004		
11	tFPG	rs10496802	2	139478604	0.0172	0.00004		
12	FPG	rs7684538	4	96725483	0.0202	0.00005	UNC5C	
13	Fasting Insulin	rs963328	1	209426056	0.2949	0.00005	FLVCR	
14	Incident DM	rs2432961	8	120266196	0.0069	0.00005		
15	Incident DM	rs2468168	8	120238819	0.0426	0.00006	COLEC10	
16	ISI_0-120	rs6594987	5	116256211	0.1298	0.00006		
17	HOMA-IR	rs10511885	9	31821043	0.1250	0.00007		
18	tFPG	rs10487976	7	122429976	0.0076	0.00007	SLC13A1	
19	tFPG	rs2204295	7	122432041	0.0060	0.00007	SLC13A1	
20	HbA1c	rs9325002	5	145406441	0.0295	0.00007	SH3RF2	
21	HbA1c	rs1365371	8	129018405	0.0298	0.00007		
22	HOMA-IR	rs2020362	19	36033107	0.0016	0.00009		
23	Incident DM	rs1489092	3	76204196	0.0535	0.00010		
24	ISI_0-120	rs2942321	5	19365227	0.4753	0.00010		
25	ISI_0-120	rs10501828	11	94883857	0.0067	0.00010		

**2c. LOD > 2, Ordered by Lod Score**								

**No.**	**Trait**	**SNP or STR**	**Chr**	**Physical position**	**Marshfield cM**	**Max LOD**	**Physical position**	
							
							**Lower bound where LOD = 1.5**	**Upper bound where LOD = 1.5**

1	FPG	rs1890843	1	207225242	230.9	3.64	205357346	209935673
2	HbA1c	rs1463697	3	195278503	217.5	3.16	191762568	197963623
3	HOMA-IR	rs10513843	3	190998205	209.4	3.08	188644318	193634077
4	Fasting insulin	rs4803953	19	51650847	70.1	2.98	43726908	56203682
5	HbA1c	rs10510060	10	121853460	139.9	2.41	119524854	125827901
6	HOMA-IR	rs10500300	19	53439815	73.3	2.36	42063572	57117898
7	tFPG	rs2837076	21	39850406	38.7	2.36	34879124	41000937
8	HbA1c	rs10497392	2	174176465	177.5	2.30	153549351	177629785
9	tFPG	rs876362	2	80327060	102.6	2.29	70585709	112151653
10	Fasting insulin	rs10513860	3	191902243	212.8	2.21	187447466	196384998
11	FPG	rs1882347	2	164233821	167.3	2.20	146837297	171264176
12	FPG	rs10512296	9	102073227	108.4	2.15	91062454	107815119
13	tFPG	ATA20G07	5	180431	0.0	2.10	180431	2855065
14	tFPG	rs2444962	15	31214059	24.7	2.05	23732660	35462954
15	HbA1c	rs10494382	1	159838765	175.2	2.03	157467831	200055293

The FHS has multiple measures of diabetes-related quantitative traits. We used a multiple-related trait approach in a strategy different from prioritizing SNPs based solely on small p-values. This approach yielded 203 SNPs associated with multiple traits. Of these, 53 were also associated with incident diabetes (p < 0.01 by GEE or FBAT). We defined redundant SNPs as those in LD with *r*^2 ^>= 0.80 to select 168 non-redundant SNPs associated with multiple traits; 42 of these non-redundant SNPs also were associated with incident diabetes (Table 3). Examination of the multiple trait-based approach revealed 1) consistent associations of traits with SNPs that were in LD (providing reassurance that the signal was due to an association of traits with a particular genomic region rather than to technical error); 2) several putative associations of traits with SNPs in the same gene but not in perfect LD (suggesting that the association signal may be due to a functional role of that gene rather than a statistical fluctuation); and 3) associations of traits with SNPs in a variety of novel but plausible biological candidate genes.

**Table 3:** Forty two (42) SNPs associated with (FPG, HbA1c, and tFPG) OR (fasting insulin, HOMA-IR, and ISI_0-120) OR (any two of either) AND incident DM

No.	Chr	SNP	N other SNPs with r^2 ^> 0.8	Minor Allele A/G/T/C	MAF	Gene *	Gene Position	GEE Mean p-value		FBAT Mean p-value		Cox p-value	Minor Allele Cox HR for DM	FBAT DM Incidence p-value
											
								3 Glucose Traits	3 Insulin Traits	3 Glucose Traits	3 Insulin Traits			
1	12	rs1368254	135	G	48.3%	*LOC387882*	Near	0.02	0.001	0.01	0.007	0.007	0.67	0.0008
2	12	rs10506806	76	T	29.7%		Out	0.003	0.03	0.01	0.01	0.02	0.65	0.004
3	2	rs10496417	74	A	34.5%	*SLC5A7*	Near	0.01	0.003	0.04	0.02	0.007	1.58	0.11
4	8	rs10503835	8	C	21.9%	*HMBOX1*	In	0.004	0.03	0.03	0.008	0.005	0.59	0.03
5	5	rs459743	83	C	16.9%		Out	0.009	0.009	0.03	0.02	0.002	0.42	0.012
6	10	rs1879316	55	A	13.5%	*RASGEF1A*	Near	0.004	0.001	0.18	0.07	0.009	0.45	0.48
7	13	rs2066219	79	G	23.0%		Out	0.005	0.0009	0.22	0.08	0.009	0.59	0.22
8	7	rs10487974	11	A	36.9%	*SLC13A1*	Near	0.02	0.08	0.002	0.02	0.001	0.58	0.001
9	3	rs1878175	54	G	11.2%		Out	0.003	0.003	0.39	0.02	0.003	0.36	0.12
10	3	rs697957	32	T	25.2%	*CD47*	Near	0.005	0.03	0.03	0.03	0.001	1.65	0.02
11	1	rs952635	9	G	31.3%	*PDE4B*	In	0.0007	0.009	0.06	0.41	0.001	0.56	0.16
12	3	rs10512839	77	C	25.9%	*CPNE4*	In	0.02	0.02	0.05	0.009	0.000	1.78	0.03
13	12	rs4767161	84	A	13.3%	*RBM19*	In	0.19	0.05	0.01	0.002	0.15	0.63	0.0014
14	2	rs1073893	27	A	17.7%	*FLJ32745*	In	0.008	0.16	0.003	0.06	0.18	0.75	0.0008
15	16	rs10500547	133	G	16.3%	*AB051533*	In	0.07	0.06	0.03	0.003	0.21	1.29	0.002
16	3	rs1489100	29	G	41.0%		Out	0.01	0.06	0.004	0.16	0.09	0.75	0.0015
17	2	rs2367204	73	G	47.4%	*IMMT*	In	0.007	0.006	0.27	0.05	0.001	1.58	0.03
18	4	rs10489088	71	C	12.7%		Out	0.24	0.22	0.01	0.001	0.35	1.22	0.005
19	20	rs6093416	86	A	14.0%	*TOP1*	Near	0.01	0.003	0.22	0.12	0.00	0.35	0.02
20	5	rs871853	14	G	41.5%	*CPLX2*	In	0.10	0.68	0.001	0.02	0.36	1.15	0.0003
21	7	rs1355037	156	C	23.4%	*ZPBP*	In	0.29	0.07	0.05	0.001	0.32	1.18	0.006
22	8	rs4418368	82	T	36.1%	*DLGAP2*	In	0.08	0.08	0.03	0.008	0.27	1.18	0.004
23	4	rs10516471	26	G	43.6%	*PPP3CA*	In	0.006	0.12	0.02	0.10	0.04	0.70	0.007
24	17	rs2322969	158	C	46.6%		Out	0.18	0.006	0.08	0.02	0.04	1.34	0.003
25	4	rs1395114	28	A	19.3%	*BX537758*	In	0.004	0.17	0.01	0.22	0.001	1.79	0.02
26	7	rs711517	88	G	18.4%		Out	0.02	0.008	0.26	0.05	0.000	1.86	0.17
27	10	rs332148	80	T	18.5%	*WAC*	In	0.02	0.01	0.18	0.09	0.004	0.46	0.03
28	16	rs2042389	136	T	32.9%		Out	0.10	0.17	0.07	0.002	0.003	1.54	0.05
29	16	rs7186570	90	G	17.2%	*A2BP1*	In	0.34	0.12	0.01	0.008	0.91	1.03	0.005
30	8	rs9297181	36	A	19.4%		Out	0.18	0.08	0.004	0.08	0.25	0.79	0.003
31	18	rs540128	85	A	36.2%	*PHLPP*	In	0.38	0.13	0.02	0.005	0.83	0.97	0.004
32	14	rs1954673	78	A	26.4%		Out	0.005	0.008	0.35	0.43	0.005	0.56	0.63
33	10	rs10509923	154	C	33.0%	*CSPG6*	Near	0.18	0.002	0.42	0.03	0.008	0.64	0.20
34	5	rs861085	35	T	30.6%	*NUDT12*	Near	0.008	0.48	0.01	0.28	0.001	0.52	0.02
35	1	rs7531174	33	C	20.6%	*SLC44A3*	In	0.001	0.21	0.09	0.68	0.001	1.72	0.09
36	7	rs6949530	57	T	18.8%	*TAS2R16*	Near	0.67	0.83	0.007	0.005	0.96	0.99	0.009
37	5	rs2967017	137	T	45.1%		Out	0.65	0.15	0.06	0.003	0.82	1.04	0.007
38	3	rs729511	159	A	43.8%	*SLC9A9*	In	0.45	0.003	0.14	0.13	0.03	0.70	0.008
39	9	rs1060586	155	T	49.3%	*RBM18*	Near	0.16	0.0008	0.67	0.28	0.00	1.60	0.99
40	17	rs2190706	157	T	34.2%		Out	0.48	0.04	0.20	0.007	0.00	1.55	0.03
41	3	rs509208	31	C	16.8%		Out	0.002	0.06	0.64	0.53	0.01	0.51	0.63
42	10	rs7089102	87	G	47.2%		Out	0.04	0.006	0.71	0.49	0.01	1.48	0.86

* Gene symbol and position from UCSC Genome Browser (http://genome.ucsc.edu/; accessed September 2006); SNPs within 60 kb of a known gene are considered 'Near'.

We used the UCSC Genome Browser (http://genome.ucsc.edu/; accessed September 2006) to annotate SNP details [30,31]. Of the 823 (736 + 203; 116 overlapped) SNPs identified by both prioritization methods without removing SNPs in LD (*r*^2 ^>= 0.80), 304 (36.9%) were in genes, 173 (21%) were within 60 kb of a known gene and 5 (0.61%) were coding. For comparison, of the 70,987 SNPs included in this analysis, 25,916 (36.5%) were in genes, 14,333 (20.2%) were within 60 kb of a known gene and 421 (0.59%) were coding.

Some SNPs had p-values < 0.001 overlapping more than one analytical method. For instance, 18 SNPs were associated at p < 0.001 with at least one quantitative trait in both the GEE and the FBAT analyses. For incident diabetes, 5 SNPs were associated with diabetes survival in the Cox models and with age-of-onset in the FBAT analyses.

We used the FHS 100K array data to verify, *in silico*, replicated associations of reported diabetes candidate genes (Table 4). We found 7 SNPs in or near *TCF7L2*. One 100K SNP (rs7100927) was in moderate LD (r^2 ^= 0.5) with *TCF7L2*-associated SNP rs7903146 and was nominally associated with a 56% increased relative risk of diabetes (p = 0.007) and with tFPG (GEE p = 0.03). We found 6 SNPs in or near *ABCC8*, but no SNPs in strong LD with *ABCC8 *A1369S (rs757110) or *KCNJ11 *E23K (rs5219), and thus could not replicate these associations. One 100K SNP (rs878208) ~25 kb upstream of *ABCC8 *showed nominal association with risk of diabetes, but it was not in LD with rs757110 in *ABCC8 *(r^2 ^= 0.04). We found 15 SNPs in or near *PPARG*, but none were associated with diabetes. Four SNPs were associated (p < 0.05) with quantitative traits but were not in LD (r^2 ^< 0.03) with *PPARG *P12A (rs1801282), the variant previously associated with type 2 diabetes [7]. We found no polymorphic (MAF > 1%) 100K SNPs in, near, or in LD with *CAPN10 *or *HNFA*.

**Table 4:** FHS 100K SNP Test of Association with SNPs in Established Candidate Genes for Type 2 Diabetes

Candidate Gene	Candidate SNP	Physical Position	FHS 100K SNP	Physical Position	**r**^2^	GEE lowest p-value	GEE Trait	FBAT Lowest p-value	FBAT Trait	Cox p-value	Cox HR for DM
***ABCC8***	rs757110	17375053	rs878208	17478662	0.04	**0.05**	Fasting insulin	0.12	Fasting insulin	**0.02**	1.96
			rs722341	17429722	0.05	0.11	tFPG	**0.009**	HbA1c	0.70	1.09
			rs916829	17397049	0.02	0.18	Fasting insulin	**0.02**	Fasting insulin	0.47	0.84
			rs2283257	17446021	0.03	0.23	ISI_0-120	0.05	ISI_0-120	0.98	0.99
			rs2299641	17397566	0.01	0.38	Fasting insulin	0.21	ISI_0-120	0.19	1.24
			rs2190454	17490211	0.01	0.35	Fasting insulin	0.25	ISI_0-120	0.48	0.90
***PPARG***	rs1801282	12368125	rs10510422	12505413	0.00	**0.003**	tFPG	0.13	ISI_0-120	0.13	3.86
			rs3856808	12505184	0.00	**0.005**	tFPG	0.17	ISI_0-120	0.11	0.25
			rs10510421	12502242	0.00	**0.006**	tFPG	0.14	ISI_0-120	0.12	0.26
			rs2938392	12409608	0.03	**0.007**	Fasting insulin	0.14	Fasting insulin	0.36	1.13
			rs709157	12526881	0.00	0.05	ISI_0-120	0.10	ISI_0-120	0.68	0.93
			rs10510418	12363563	0.04	0.07	Fasting insulin	0.10	ISI_0-120	0.46	0.89
			rs1801282	12368125	**1.00**	0.07	ISI_0-120	0.20	HbA1c	0.89	0.96
			rs1899951	12369840	**1.00**	0.11	ISI_0-120	0.25	HbA1c	0.86	0.96
			rs4135268	12525199	0.01	0.11	ISI_0-120	0.20	tFPG	0.80	0.92
			rs10510417	12352294	0.31	0.17	ISI_0-120	0.45	ISI_0-120	0.62	0.91
			rs2292101	12409901	0.00	0.19	tFPG	0.22	Fasting insulin	0.08	1.62
			rs10510419	12401936	0.01	0.26	tFPG	0.35	ISI_0-120	0.24	1.31
			rs10510410	12321738	0.31	0.38	FPG	0.36	Fasting insulin	0.88	0.97
			rs10510411	12321849	0.31	0.40	FPG	0.39	Fasting insulin	0.94	0.99
			rs10510412	12321962	0.31	0.44	FPG	0.38	Fasting insulin	0.84	1.03
	rs12255372*	114798892	rs10509967	114685922	0.00	**0.04**	HbA1c	0.12	ISI_0-120	0.82	0.96
***TCF7L2***	rs7903146*	114748339	**rs7100927**	**114786038**	**0.50**	**0.03**	**tFPG**	**0.13**	**tFPG**	**0.007**	**1.56**
			rs10509966	114666170	0.00	**0.04**	HbA1c	0.07	ISI_0-120	0.64	1.09
			rs10509969	114903549	0.08	0.14	Fasting insulin	0.08	FPG	0.60	0.89
			rs290483	114905204	0.10	0.17	Fasting insulin	0.34	tFPG	0.93	0.99
			rs7917983	114722872	0.09	0.27	tFPG	0.29	HbA1c	0.17	0.82
			rs10509970	114904903	0.05	0.32	tFPG	0.43	tFPG	0.51	1.14

* LD betweaen rs12255372 and rs7903146 in HapMap CEU: *r*^2 ^= 0.78; Bold = r^2 ^>= 0.5 or p-value < 0.05

We also assessed our approach for confirmation of 4 SNPs associated with FPG reported on the Boston University Department of Genetics and Genomics public site http://gmed.bu.edu/about/index.html that displays selected associations with FHS 100K data. We found no association (all p-values > 0.6) of incident diabetes or levels of FPG with SNPs rs10495355, rs9302082, rs10483948, or rs1148509.

## Discussion and conclusion

In this paper we describe the characteristics and initial GWA results for type 2 diabetes and related quantitative traits in the FHS 100K SNP resource. Over 1000 men and women from a community-based sample have detailed linkage and association of diabetes-related phenotypes and 100K dense array SNP results available on the web. About 0.3%–0.6% of SNPs in the 100K array with MAF > 10% are associated at p < 0.001 with six diabetes-related quantitative traits or with incident type 2 diabetes. A similar proportion of SNPs in the array (0.21%) are associated with multiple related diabetes traits. These several hundred SNPs likely contain more false positive than true positive associations with diabetes and related traits, however, they offer logical next targets for the follow-up replication studies in independent samples necessary to resolve true diabetes risk genes. The FHS 100K data replicate the otherwise widely-replicated *TCF7L2 *association with diabetes [11,12,32-40] in an *in silico *analysis.

The FHS 100K SNP data resource has potential value to detect and replicate novel type 2 diabetes susceptibility genes. The 100K SNP array is limited by relatively sparse coverage in some regions, accounting on average for just 30%–40% of the human genome in whites [17,41]. Association with the risk SNP in *TCF7L2 *is detectable at p < 0.05, but there are no SNPs in adequate LD with *ABCC8 *or *PPARG *to assess replication of causal SNPs in these accepted diabetes susceptibility genes. Thin coverage will be remedied to a large degree by the incipient availability in FHS of Affymetrix 500 k SNP array data as part of the planned FHS SHARe Study. (http://www.nhlbi.nih.gov/meetings/nhlbac/sept06sum.htm; accessed September 2006) Our analysis also demonstrates that true positive diabetes susceptibility gene signals are likely to be associated with modest p-values and will remain challenging to detect at the stringent p-values required for GWA studies. The enormous datasets generated by GWA scans have the potential to greatly advance understanding, or conversely to overwhelm the field with false leads. SNP prioritization strategies that leverage the complexity of the diabetes phenotype may offer some advantages over strictly p-value driven approaches. Replication, fine mapping, and functional studies are required to determine which approaches are most efficient and which SNPs are true positive diabetes risk factors. Integration with other GWA scans in similar cohorts will allow *in silico *replication of significant findings, increase power and reveal generalizability.

This report details the FHS contribution to publicly available diabetes-related genetic data. An important key to efficiently and economically achieving adequate power to detect association will be to integrate information from several GWA scans. While several cohorts have been assembled to perform GWA scans in type 2 diabetes, few possess the wealth of longitudinal, multigenerational phenotypic data available in Framingham. The FHS complements extant type 2 diabetes GWA studies. This report guides the way to harness the power of the FHS 100K SNP GWA resource to identify type 2 diabetes susceptibility genes.

## Abbreviations

FPG = fasting plasma glucose; FBAT = family-based association test; FHS = Framingham Heart Study; GEE = generalized estimating equations; GWA = Genome-wide association; HbA1c = hemoglobin A1c; HOMA-IR = homeostasis model insulin resistance; HWE = Hardy Weinberg equilibrium; IBD = Identity-by-descent; ISI_0-120 = 0–120 min insulin sensitivity index; LD = Linkage disequilibrium; LOD = Log odds score; MAF = Minor allele frequency; SNP = Single nucleotide polymorphism;TFPG = 28-yr time-averaged FPG.

## Authors' contributions

All authors participated in the design and conduct of the study and edited and approved the final manuscript. JM drafted the manuscript and coordinated the study. JM and CF contributed to FHS diabetes-related phenotyping. JD, AM, and and LAC coordinated the data management and conducted the statistical analyses. CL prepared traits for analyses. JF contributed the multiple-related traits method for SNP selection and the literature review for Table 4.
